# Validation of the Gail model for predicting individual breast cancer risk in a prospective nationwide study of 28,104 Singapore women

**DOI:** 10.1186/bcr3104

**Published:** 2012-01-30

**Authors:** Wen Yee Chay, Whee Sze Ong, Puay Hoon Tan, Nicholas Qi Jie Leo, Gay Hui Ho, Chia Siong Wong, Kee Seng Chia, Khuan Yew Chow, MinHan Tan, Peter Ang

**Affiliations:** 1 Department of Medical Oncology, National Cancer Centre Singapore, 11 Hospital Drive, Singapore 169610, Republic of Singapore; 2 Division of Clinical Trials and Epidemiological Sciences, National Cancer Centre Singapore, 11 Hospital Drive, Singapore 169610, Republic of Singapore; 3 Department of Pathology, Singapore General Hospital, Outram Road, Singapore 169608, Republic of Singapore; 4 Department of Surgical Oncology, National Cancer Centre Singapore, 11 Hospital Drive, Singapore 169610, Republic of Singapore; 5 Centre for Molecular Epidemiology, Yong Loo Lin School of Medicine, National University of Singapore, MD3 Level 3, 16 Medical Drive, Singapore 117597, Republic of Singapore; 6 National Registry of Diseases Office, Level 5 Health Promotion Board, 3 Second Hospital Avenue, Singapore 168937, Republic of Singapore; 7 Department of Biodevices and Diagnostics, Institute of Bioengineering and Nanotechnology, Nanos, 31 Biopolis Way, Singapore 138669, Republic of Singapore; 8 Department of Medical Oncology, OncoCare Cancer Centre, 6 Napier Road, Gleneagles Medical Centre #03-02/03-13, Singapore 258499, Republic of Singapore

## Abstract

**Introduction:**

The Gail model (GM) is a risk-assessment model used in individual estimation of the absolute risk of invasive breast cancer, and has been applied to both clinical counselling and breast cancer prevention studies. Although the GM has been validated in several Western studies, its applicability outside North America and Europe remains uncertain. The Singapore Breast Cancer Screening Project (SBCSP) is a nation-wide prospective trial of screening mammography conducted between Oct 1994 and Feb 1997, and is the only such trial conducted outside North America and Europe to date. With the long-term outcomes from this study, we sought to evaluate the performance of GM in prediction of individual breast cancer risk in a Asian developed country.

**Methods:**

The study population consisted of 28,104 women aged 50 to 64 years who participated in the SBSCP and did not have breast cancer detected during screening. The national cancer registry was used to identify incident cases of breast cancer. To evaluate the performance of the GM, we compared the expected number of invasive breast cancer cases predicted by the model to the actual number of cases observed within 5-year and 10-year follow-up. Pearson's Chi-square test was used to test the goodness of fit between the expected and observed cases of invasive breast cancers.

**Results:**

The ratio of expected to observed number of invasive breast cancer cases within 5 years from screening was 2.51 (95% confidence interval 2.14 - 2.96). The GM over-estimated breast cancer risk across all age groups, with the discrepancy being highest among older women aged 60 - 64 years (E/O = 3.53, 95% CI = 2.57-4.85). The model also over-estimated risk for the upper 80% of women with highest predicted risk. The overall E/O ratio for the 10-year predicted breast cancer risk was 1.85 (1.68-2.04).

**Conclusions:**

The GM over-predicts the risk of invasive breast cancer in the setting of a developed Asian country as demonstrated in a large prospective trial, with the largest difference seen in older women aged between 60 and 64 years old. The reason for the discrepancy is likely to be multifactorial, including a truly lower prevalence of breast cancer, as well as lower mammographic screening prevalence locally.

## Introduction

The Gail model (GM) for evaluation of the absolute individual risk of invasive breast cancer in women has been used extensively in Western populations for individual counseling and cancer prevention trial design. Given that international breast cancer incidence varies widely and that the validation of GM has been conducted predominantly in Western populations, it is relevant to examine its performance in different settings. The age-standardized rate of breast cancer was 59.5 per 100,000 persons per year among Singaporean women in the period of 2004 to 2008 [1]. This is considerably lower in comparison with the West, where the estimated age-standardized breast cancer incidence rates for 1998 through 2002 were 86.3 per 100,000 women in Italy and 76.7 per 100,000 women in the US [2]. Given these differences, it is possible that a breast cancer prediction model such as the GM, which uses a small number of risk factors to estimate an absolute risk of invasive breast cancer, estimates that are less well calibrated when applied to varying populations. Indeed, the accuracy of the calibration of the GM might depend on the extent of the prevalence of risk factors as well as differences in the use of screening mammography. In the US, 70.1% of women who were older than 39 years of age in 2000 reported having had a mammogram within the previous 2 years [3]; this figure is distinctly higher than that seen in Asian populations. For example, in Singapore, an estimated 40.9% of women between the ages of 50 to 69 years had a mammography screen in the last 2 years [4].

The original GM was derived from data of the Breast Cancer Detection Demonstration Project (BCDDP), a program consisting of five annual mammography screens across 29 centers in the US [5]. By means of multivariate relative risk components and baseline age-specific breast cancer risks estimated from the BCDDP population, Gail model 1 was formed to estimate the absolute risk of both invasive and *in situ *breast cancer. This model was subsequently modified by investigators from the Breast Cancer Prevention Trial [6] by using invasive breast cancer rates from the Surveillance, Epidemiology, and End Results (SEER) program of the National Cancer Institute (NCI) to better estimate the risks of invasive breast cancers. This modified model, referred to as 'Gail model 2' [7], has been validated by using independent data from the US and hereafter is referred to as GM in our study. Rockhill and colleagues [8] used data from the Nurses' Health Study for the period from 1992 to 1997 for calibration of the calculated average absolute breast cancer risk predicted by the GM. Overall, Rockhill and colleagues concluded that the GM was well calibrated but had only modest discriminatory accuracy at the individual level. This is consistent with the findings of Costantino and colleagues [7], who found an overall ratio of expected to observed (E/O) breast cancer cases of 1.03 for women in the control arm of the Breast Cancer Prevention Trial and also concluded that the GM was well calibrated. Interestingly, although mammographic screening for women older than 50 years has become routine practice in many Western countries, no prospective data from large studies have examined mammography in Asian women. Relatively few publications have evaluated the GM in a population outside the US; even then, these validation studies were based primarily on limited case control data, which may perform better for assessment of relative rather than absolute risk [9-11].

From 1968 to 2007, Singapore experienced an almost threefold increase in breast cancer incidence. The incidence rate of breast cancer from 2003 to 2007 was 2.9 times that in 1968 to 1972 and 5.65% higher than in 1998 to 2002 [12]. Our study was undertaken to evaluate the utility of the GM in an Asian population by using 10-year follow-up data from the Singapore Breast Cancer Screening Project (SBCSP), which is a large-scale prospective community mammography screening program. To the best of our knowledge, ours is the first study evaluating the systemic application and validation of the GM by using long-term outcomes in a prospectively studied Asian population. The goal was to evaluate the performance of the GM as an appropriate breast cancer risk assessment tool in the Asian population.

## Materials and methods

### Singapore breast cancer screening project

The SBCSP was a population-based mammography screening project conducted between October 1994 and February 1997 among female Singaporeans. The design of the SBCSP has been described in detail previously [13]. Briefly, all women who were 50 to 64 years old on 1 October 1994 were first identified from a comprehensive population registry (*n *= 166,600). A total of 69,473 (41.7%) of these women were randomly selected and invited for a single free mammography screening from 1 October 1994 to 28 February 1997. Prior to mammography, all attendees completed a questionnaire to determine eligibility, and only those who were eligible proceeded to further interview and mammography. Women who had cancers of the breast or other sites (except non-melanoma skin cancer) and had mammography or breast biopsy in the 12 months prior to screening or were pregnant were excluded from screening. In total, 28,235 women from 50 to 64 years of age participated in this initial mammography screening program with a single two-view mammogram examination. No subsequent free mammography screening was offered.

### Study population

Among the 28,235 participants, 131 women were detected with breast cancer during initial screening and were excluded from the study population. Therefore, a total of 28,104 women provided the basis of this study. Electronic matching with the Singapore Cancer Registry and the National Death Register was used to identify any breast cancers occurring among these women from the date of the screen until December 2007. Permission was obtained from the Ministry of Health (Singapore) and the Ministry of Home Affairs (Singapore), respectively, for data access. One hundred forty-four and 409 invasive breast cancers were diagnosed within 5 years and 10 years from screening, respectively. Women not diagnosed with breast cancer during each period formed the corresponding control group for comparison with the diagnosed cases.

### Formation of Gail model risk factor categories

Risk factor categories in the GM were formed by using information from the screening questionnaire: age at menarche (at least 14 years, 12 to 13 years, or fewer than 12 years), age at first live birth (nulliparous, fewer than 20 years, 20 to 24 years, 25 to 29 years, or at least 30 years), previous breast biopsy, and number of first-degree relatives with breast cancer. Number of previous breast biopsies and presence of atypical hyperplasia in biopsy were not ascertained in the questionnaire, which asked only whether women had had any previous breast biopsy. The questionnaire asked each woman whether she had a mother, any sister(s), or any daughter(s) with breast cancer and not the actual number of affected first-degree relatives. Assuming that women who reported that they had only sister(s) or only daughter(s) with breast cancer were recorded as having one affected first-degree relative, we recategorized our information on family history to three categories of 0, 1, or 2 or more affected first-degree relatives.

### Statistical methods

The 5-year and 10-year absolute risks of each individual woman were calculated from their age of screening until end of follow-up at 5 years and 10 years, respectively. The calculation was done by using publicly available SAS codes for GM prediction from the NCI's Breast Cancer Risk Assessment Tool website [14]. The expected number of cases (E) among women in a category was calculated by summing the estimated absolute risk across all women in the category.

Model calibration was assessed by comparison of the expected (E) and observed (O) number of breast cancer cases by GM risk factor categories, 5-year age group, and quintiles of predicted risk. Quintiles were formed by ranking all women by their predicted risk from the GM in ascending order. Confidence intervals (CIs) were calculated for E/O ratio on the basis of exact theory, and O was assumed to follow a Poisson distribution. The Pearson chi-squared test was used to test the goodness of fit between E and O. Comparison of categorical variables between diagnosed cases and the control group was performed by using the chi-squared test or Fisher exact test as appropriate. A *P *value of less than 0.05 was considered statistically significant. All analyses were performed by using SAS 9.1 (SAS Institute Inc., Cary, NC, USA).

Matsuno and colleagues [15] recently reported the creation and use of an Asian American Breast Cancer Study (AABCS) model that was based on data from the AABCS combined with use of ethnicity-specific data from the NCI's SEER program. This model was calibrated by using data from the Asia Pacific women in the Women's Health Initiative. Using this model, the authors reported lower rates of breast cancer incidence in Chinese-American women compared with Caucasian-American women. Applying this AABCS model to the SBCSP population, we attempted to predict the breast cancer risk by using the Chinese-American AABCS model for the SBCSP Chinese women and the other Asian-American AABCS model for Malays, Indians, and other racial groups in the SBCSP.

## Results

Up to December 2007, 575 of 28,104 individuals developed ductal carcinoma *in situ *(DCIS) invasive breast cancer. The distribution of the study cohort by risk factors in the GM is shown in Table 1. There were proportionally more breast cancer cases with early menarche, previous breast biopsy, a late age at first childbirth, and at least one first-degree relative with breast cancer than the controls in the SBCSP study cohort.

**Table 1:** Distribution of Singapore Breast Cancer Screening Project cohort by risk factors in Gail model

	Total		Cases (all breast cancer)		Controls		*P *value
	Number	Percentage	Number	Percentage	Number	Percentage	
Total	28,104	100.0	575	100.0	27,529	100.0	
Age at menarche, years							
≥ 14	18,237	64.9	324	56.3	17,913	65.1	< 0.001
12-13	8,952	31.9	213	37.0	8,739	31.7	
< 12	915	3.3	38	6.6	877	3.2	
Any breast biopsy Age at screening: ≥50 years							
No	26,631	94.8	514	89.4	26,117	94.9	< 0.001
Yes	1,473	5.2	61	10.6	1,412	5.1	
Number of affected first-degree relatives Age at first live birth: < 20 years							
0	4,753	16.9	68	11.8	4,685	17.0	< 0.001^b^
1	89	0.3	3	0.5	86	0.3	
> 1	1	0.0	0	0.0	1	0.0	
Number of affected first-degree relatives Age at first live birth: 20-24 years							
0	10,321	36.7	149	26.3	10,172	37.0	
1	216	0.8	13	2.1	203	0.7	
> 1	6	0.0	2	0.3	4	0.0	
Number of affected first-degree relatives Age at first live birth: 25-29 years or nulliparous							
0	9,246	32.9	230	40.0	9,016	32.8	
1	303	1.1	17	3.0	286	1.0	
> 1	4	0.0	0	0.0	4	0.0	
Number of affected first-degree relatives Age at first live birth: > 30 years							
0	3,059	10.9	85	14.8	2,974	10.8	
1	105	0.4	7	1.2	98	0.4	
> 1	1	0.0	1	0.2	0	0.0	

^a^All *P *values were calculated by using chi-squared test unless otherwise stated. ^b^*P *value calculated by using Fisher exact test.

The various breast cancer risk factors had a similar influence on the incidence of both DCIS and invasive breast cancers as compared with the corresponding estimated impact on risks as calculated by the GM (Table 2). However, the relative risk for each risk factor was larger on the basis of SBCSP data as compared with the GM. Women with early menarche, women with a previous breast biopsy, and women with age of first live birth after 30 years of age appeared to have a much higher risk of breast cancer. The GM provides greatest overestimation of risk in women who had late menarche, women who had no affected first-degree relative, and women who were more than 50 years old and had no prior breast biopsy at screening.

**Table 2:** Relative risks of total breast cancers based on Singapore Breast Cancer Screening Project cohort by risk factors in Gail model

Risk factors	Based on SBCSP (95% CI)	Gail model: BCDDP (95% CI)
Age at menarche, years		
≥14	1.00 (referent)	1.00 (referent)
12-13	1.39	1.10
< 12	1.95	1.21
Any breast biopsy Age at screening: ≥50 years		
No	1.00 (referent)	1.00 (referent)
Yes	1.97	1.28^a^
Number of affected first-degree relatives Age at first live birth: < 20 years		
0	1.00 (referent)	1.00 (referent)
1	3.61	2.61
> 1	13.04	6.80
Number of affected first-degree relatives Age at first live birth: 20-24 years		
0	1.33	1.24
1	4.14	2.68
> 1	12.91	5.78
Number of affected first-degree relatives Age at first live birth: 25-29 years or nulliparous		
0	1.76	1.55
1	4.75	2.76
> 1	12.79	4.91
Number of affected first-degree relatives Age at first live birth: > 30 years		
0	2.34	1.93
1	5.44	2.83
> 1	12.67	4.17

^a^Derived from Decarli and colleagues [23] (2006). BCDDP, Breast Cancer Detection Demonstration Project; CI, confidence interval; SBCSP, Singapore Breast Cancer Screening Project.

A parallel comparison between the observed breast cancers developed in the 5 years after screening for categories defined by breast cancer risk factors and the calculated expected numbers of invasive breast cancers predicted by using GM's 5-year predicted risk for Caucasian women was performed (Table 3). Overall, 362 invasive breast cancers were expected and 144 were observed. This corresponds to a ratio of expected to observed cases (E/O) of 2.51 (95% CI 2.14 to 2.96), indicating that the GM predicted a 2.51-fold higher risk of breast cancer incidence as compared with that observed over this period. Over-prediction of the number of breast cancers by GM was higher for women who were 60 to 64 years old (E/O ratio = 3.53) than for their younger counterparts who were 50 to 59 years old (E/O ratio = 2.15) (Table 4). The E/O ratios were fairly similar across the 5-year predicted risk quintile groups. Breakdown by 5-year age group and 5-year predicted risk quintile group further emphasized the over-prediction of the GM prior as compared with our risk population in certain subpopulations. The E/O ratio was greater than 5 for women who were 60 to 64 years old in the 41% to 80% quintile group.

**Table 3:** Ratios of expected (E) to observed (O) numbers of invasive breast cancers diagnosed within 5 years from screening

Risk factors	Based on Gail model (Caucasian)				Based on Matsuno AABCS model (Chinese-American and other Asian-American)			
	O	E	E/O	95% CI for E/O	O	E	E/O	95% CI for E/O
Total	144	362.08	2.51	(2.14, 2.96)	144	205.95	1.43	(1.21, 1.68)
Age at menarche, years								
≥ 14	89	228.92	2.57	(2.09, 3.17)	89	129.13	1.45	(1.18, 1.79)
12-13	47	119.66	2.55	(1.91, 3.39)	47	69.23	1.47	(1.11, 1.96)
< 12	8	13.50	1.69	(0.84, 3.37)	8	7.60	0.95	(0.48, 1.90)
	*χ*^2 ^= 131.88, *P *< 0.001				*χ*^2 ^= 19.63, *P *< 0.001			
								
Any breast biopsy Age at screening: ≥50 years								
No	129	337.48	2.62	(2.20, 3.11)	129	186.43	1.45	(1.22, 1.72)
Yes	15	24.60	1.64	(0.99, 2.72)	15	19.53	1.30	(0.78, 2.16)
	*χ*^2 ^= 132.54, *P *< 0.001				*χ*^2 ^= 18.74, *P *< 0.001			
Number of affected first-degree relatives Age at first live birth: < 20 years								
0	17	44.89	2.64	(1.64, 4.25)	17	21.22	1.25	(0.78, 2.01)
1	0	2.23	-	-	0	0.90	-	-
> 1	0	0.07	-	-	0	0.01	-	-
Number of affected first-degree relatives Age at first live birth: 20-24 years								
0	39	118.36	3.03	(2.22, 4.15)	39	62.73	1.61	(1.18, 2.20)
1	4	5.27	1.32	(0.49, 3.51)	4	2.98	0.75	(0.28, 1.99)
> 1	0	0.30	-	-	0	0.08	-	-
Number of affected first-degree relatives Age at first live birth: 25-29 years or nulliparous								
0	54	127.92	2.37	(1.81, 3.09)	54	76.46	1.42	(1.08, 1.85)
1	3	7.54	2.51	(0.81, 7.79)	3	5.71	1.90	(0.61, 5.90)
> 1	0	0.18	-	-	0	0.07	-	-
Number of affected first-degree relatives Age at first live birth: > 30 years								
0	24	52.62	2.19	(1.47, 3.27)	24	33.19	1.38	(0.93, 2.06)
1	2	2.68	1.34	(0.34, 5.36)	2	2.58	1.29	(0.32, 5.16)
> 1	1	0.04	0.04	(0.01, 0.28)	1	0.04	0.04	(0.01, 0.28)
	*χ*^2 ^= 157.85, *P *< 0.001				*χ*^2 ^= 44.82, *P *< 0.001			

AABCS, Asian American Breast Cancer Study model; CI, confidence interval; E, expected number of breast cancer cases; E/O, ratio of expected to observed number of breast cancer cases; O, observed number of breast cancer cases.

Foot note: Ratios of expected (E) numbers of invasive breast cancers using 5-year predicted risk based on Gail model and Matsuno model to observed (O) numbers of invasive breast cancers diagnosed within 5 years from screening among Singapore Breast Cancer Screening Project cohort by risk factors in Gail model

**Table 4:** Ratios of expected (E) to observed (O) numbers of invasive breast cancers by age at screening group and 5-year predicted risk quintile group

	Based on Gail model (Caucasian)				Based on Matsuno AABCS model (Chinese-American and other Asian-American)			
	O	E	E/O	95% CI for E/O	O	E	E/O	95% CI for E/O
Total	144	362.08	2.51	(2.14, 2.96)	141	205.95	1.46	(1.24, 1.72)
5-year age at screening group								
50-54 years	44	94.48	2.15	(1.60, 2.89)	44	64.39	1.46	(1.09, 1.97)
55-59 years	62	133.36	2.15	(1.68, 2.76)	62	79.48	1.28	(1.00, 1.64)
60-64 years^a^	38	134.23	3.53	(2.57, 4.85)	38	60.07	1.58	(1.15, 2.17)
	*χ*^2 ^= 134.14, *P *< 0.001				*χ*^2 ^= 18.41, *P *< 0.001			
5-year predicted risk group^b^								
1%-20%	20	49.83	2.49	(1.61, 3.86)	16	25.16	1.57	(0.96, 2.57)
21%-40%	27	60.63	2.25	(1.54, 3.27)	19	32.31	1.70	(1.08, 2.67)
41%-60%	29	69.25	2.39	(1.66, 3.44)	29	37.64	1.30	(0.90, 1.87)
61%-80%	23	78.41	3.41	(2.27, 5.13)	29	45.24	1.56	(1.08, 2.24)
81%-100%	45	103.96	2.31	(1.72, 3.09)	51	65.60	1.29	(0.98, 1.69)
	*χ*^2 ^= 132.50, *P *< 0.001				*χ*^2 ^= 19.88, *P *= 0.001			
5-year age at screening group, 5-year predicted risk group^b^								
50-54 years								
1%-20%	10	28.00	2.80	(1.51, 5.20)	2	7.07	3.54	(0.88, 14.13)
21%-40%	8	21.01	2.63	(1.31, 5.25)	5	6.02	1.20	(0.50, 2.89)
41%-60%	13	20.22	1.56	(0.90, 2.68)	10	13.68	1.37	(0.74, 2.54)
61%-80%	7	13.4	1.91	(0.91, 4.02)	10	14.62	1.46	(0.79, 2.72)
81%-100%	6	11.86	1.98	(0.89, 4.40)	17	22.99	1.35	(0.84, 2.18)
								
55-59 years								
1%-20%	7	17.96	2.57	(1.22, 5.38)	6	8.46	1.41	(0.63, 3.14)
21%-40%	14	26.54	1.90	(1.12, 3.20)	9	8.43	0.94	(0.49, 1.80)
41%-60%	11	22.76	2.07	(1.15, 3.74)	12	15.52	1.29	(0.73, 2.28)
61%-80%	10	31.77	3.18	(1.71, 5.90)	14	19.48	1.39	(0.82, 2.35)
81%-100%	20	34.33	1.72	(1.11, 2.66)	21	27.59	1.31	(0.86, 2.02)
								
60-64 years^a^								
1%-20%	3	3.85	1.28	(0.41, 3.98)	8	9.62	1.20	(0.60, 2.40)
21%-40%	5	13.09	2.62	(1.09, 6.29)	5	17.86	3.57	(1.49, 8.58)
41%-60%	5	26.27	5.25	(2.19, 12.62)	7	8.44	1.21	(0.57, 2.53)
61%-80%	6	33.24	5.54	(2.49, 12.33)	5	11.14	2.23	(0.93, 5.35)
81%-100%	19	57.78	3.04	(1.94, 4.77)	13	15.01	1.15	(0.67, 1.99)
	*χ*^2 ^= 138.51, *P *< 0.001				*χ*^2 ^= 25.92, *P *= 0.027			

^a^60-64 years group included some older than 64 years as they are screened in 1996-1997. ^b^Based on ranking of the study cohort by 10-year predicted risk in ascending order. AABCS, Asian American Breast Cancer Study model; CI, confidence interval; E, expected number of breast cancer cases; E/O, ratio of expected to observed number of breast cancer cases; O, observed number of breast cancer cases.

Ratios of expected (E) numbers of invasive breast cancers using 5-year predicted risk based on Gail model and Matsuno model to observed (O) numbers of invasive breast cancers diagnosed within 5 years from screening among Singapore Breast Cancer Screening Project cohort by age at screening group and 5-year predicted risk quintile group

Four hundred nine breast cancers developed in the 10 years after screening as compared with the 758 expected cancer cases predicted by using the GM (Table 5). The extent of over-prediction of the number of invasive breast cancers by GM within 10 years from screening (E/O ratio = 1.85) was smaller than that for the 5-year period (E/O ratio = 2.51). Over-prediction of the number of breast cancers by GM was again higher for women who were 60 to 64 years old (E/O ratio = 2.54) than for their younger counterparts who were 50 to 59 years old (E/O ratio = 1.61). Trends of E/O ratio by age group and predicted risk quintile group which were based on 10-year prediction were broadly similar to those based on 5-year prediction (Table 6).

**Table 5:** Ratios of expected (E) to observed (O) numbers of invasive breast cancers diagnosed within 10 years from screening

Risk factors	Based on Gail model (Caucasian)				Based on Matsuno AABCS model (Chinese-American and other Asian-American)			
	O	E	E/O	95% CI for E/O	O	E	E/O	95% CI for E/O
Total	409	758.18	1.85	(1.68, 2.04)	409	404.70	0.99	(0.90, 1.09)
Age at menarche, years								
≥14	226	477.32	2.11	(1.85, 2.41)	226	253.58	1.12	(0.98, 1.28)
12-13	161	252.42	1.57	(1.34, 1.83)	161	136.16	0.85	(0.72, 0.99)
< 12	22	28.44	1.29	(0.85, 1.96)	22	14.96	0.68	(0.45, 1.03)
	*χ*^2 ^= 166.89, *P *< 0.001				*χ*^2 ^= 10.84, *P *= 0.004			
Any breast biopsy Age at screening: ≥50 years								
No	370	706.46	1.91	(1.72, 2.11)	370	366.38	0.99	(0.89, 1.10)
Yes	39	51.72	1.33	(0.97, 1.82)	39	38.32	0.98	(0.72, 1.34)
	*χ*^2 ^= 163.37, *P *< 0.001				*χ*^2 ^= 0.05, *P *= 0.827			
Number of affected first-degree relatives Age at first live birth: < 20 years								
0	48	93.42	1.95	(1.47, 2.58)	48	41.62	0.87	(0.65, 1.15)
1	2	4.59	2.30	(0.57, 9.18)	2	1.76	0.88	(0.22, 3.52)
> 1	0	0.13	-	-	0	0.02	-	-
Number of affected first-degree relatives Age at first live birth: 20-24 years								
0	113	246.74	2.18	(1.82, 2.63)	113	123.47	1.09	(0.91, 1.31)
1	9	10.97	1.22	(0.63, 2.34)	9	5.84	0.65	(0.34, 1.25)
> 1	2	0.62	0.31	(0.08, 1.24)	2	0.15	0.08	(0.02, 0.30)
Number of affected first-degree relatives Age at first live birth: 25-29 years or nulliparous								
0	156	269.37	1.73	(1.48, 2.02)	156	150.21	0.96	(0.82, 1.13)
1	13	15.78	1.21	(0.70, 2.09)	13	11.19	0.86	(0.50, 1.48)
> 1	0	0.36	-	-	0	0.14	-	-
Number of affected first-degree relatives Age at first live birth: > 30 years								
0	62	110.49	1.78	(1.39, 2.29)	62	65.18	1.05	(0.82, 1.35)
1	3	5.61	1.87	(0.60, 5.80)	3	5.04	1.68	(0.54, 5.21)
> 1	1	0.09	0.09	(0.01, 0.64)	1	0.08	0.08	(0.01, 0.57)
	*χ*^2 ^= 179.85, *P *< 0.001				*χ*^2 ^= 38.66, *P *< 0.001			

AABCS, Asian American Breast Cancer Study model; CI, confidence interval; E, expected number of breast cancer cases; E/O, ratio of expected to observed number of breast cancer cases; O, observed number of breast cancer cases.

Ratios of expected (E) numbers of invasive breast cancers using 10-year predicted risk based on Gail model and Matsuno model to observed (O) numbers of invasive breast cancers diagnosed within 10 years from screening among Singapore Breast Cancer Screening Project cohort by risk factors in Gail model

**Table 6:** Ratios of expected (E) to observed (O) numbers of invasive breast cancers by age at screening group and 10-year predicted risk quintile group

	Based on Gail model (Caucasian)				Based on Matsuno AABCS model (Chinese-American and other Asian-American)			
	O	E	E/O	95% CI for E/O	O	E	E/O	95% CI for E/O
Total	409	758.18	1.85	(1.68, 2.04)	409	404.70	0.99	(0.90, 1.09)
5-year age at screening group								
50-54 years	135	205.49	1.52	(1.29, 1.80)	135	128.96	0.96	(0.81, 1.13)
55-59 years	168	283.71	1.69	(1.45, 1.96)	168	153.13	0.91	(0.78, 1.06)
60-64 years^a^	106	268.94	2.54	(2.10, 3.07)	106	122.58	1.16	(0.96, 1.40)
	*χ*^2 ^= 170.09, *P *< 0.001				*χ*^2 ^= 3.97, *P *= 0.137			
5-year predicted risk group^b^								
1%-20%	62	107.19	1.73	(1.35, 2.22)	49	49.33	1.01	(0.76, 1.33)
21%-40%	70	128.26	1.83	(1.45, 2.32)	55	64.54	1.17	(0.90, 1.53)
41%-60%	60	144.77	2.41	(1.87, 3.11)	82	74.29	0.91	(0.73, 1.12)
61%-80%	89	163.95	1.84	(1.50, 2.27)	86	88.32	1.03	(0.83, 1.27)
81%-100%	128	214.00	1.67	(1.41, 1.99)	137	128.21	0.94	(0.79, 1.11)
	*χ*^2 ^= 163.98, *P *< 0.001				*χ*^2 ^= 2.88, *P *= 0.579			
5-year age at screening group 5-year predicted risk group^b^								
50-54 years								
1%-20%	29	56.55	1.95	(1.36, 2.81)	8	11.85	1.48	(0.74, 2.96)
21%-40%	28	38.62	1.38	(0.95, 2.00)	13	13.00	1.00	(0.58, 1.72)
41%-60%	19	37.93	2.00	(1.27, 3.13)	35	24.69	0.71	(0.51, 0.98)
61%-80%	35	39.24	1.12	(0.80, 1.56)	28	32.03	1.14	(0.79, 1.66)
81%-100%	24	33.15	1.38	(0.93, 2.06)	51	47.37	0.93	(0.71, 1.22)
								
55-59 years								
1%-20%	21	32.73	1.56	(1.02, 2.39)	22	17.32	0.79	(0.52, 1.20)
21%-40%	35	65.44	1.87	(1.34, 2.60)	22	19.19	0.87	(0.57, 1.32)
41%-60%	21	29.51	1.41	(0.92, 2.16)	29	29.55	1.02	(0.71, 1.47)
61%-80%	36	78.42	2.18	(1.57, 3.02)	38	35.81	0.94	(0.69, 1.30)
81%-100%	55	77.60	1.41	(1.08, 1.84)	57	51.26	0.90	(0.69, 1.17)
								
60-64 years^a^								
1%-20%	12	17.87	1.49	(0.85, 2.62)	19	20.15	1.06	(0.68, 1.66)
21%-40%	7	24.20	3.46	(1.65, 7.25)	20	32.35	1.62	(1.04, 2.51)
41%-60%	20	77.33	3.87	(2.49, 5.99)	18	20.02	1.11	(0.70, 1.77)
61%-80%	18	46.29	2.57	(1.62, 4.08)	20	20.48	1.02	(0.66, 1.59)
81%-100%	49	103.25	2.11	(1.59, 2.79)	29	29.58	1.02	(0.71, 1.47)
	*χ*^2 ^= 181.57, *P *< 0.001				*χ*^2 ^= 13.81, *P *= 0.464			

^a^Group of 60- to 64-year-olds included some older than 64 years as they were screened in 1996-1997. ^b^Based on ranking of the study cohort by 10-year predicted risk in ascending order. AABCS, Asian American Breast Cancer Study model; CI, confidence interval; E, expected number of breast cancer cases; E/O, ratio of expected to observed number of breast cancer cases; O, observed number of breast cancer cases.

Ratios of expected (E) numbers of invasive breast cancers using 10-year predicted risk based on Gail model and Matsuno model to observed (O) numbers of invasive breast cancers diagnosed within 10 years from screening among Singapore Breast Cancer Screening Project cohort by age at screening group and 10-year predicted risk quintile group

## Discussion

### Use of Gail model in an Asian setting

The GM is widely used in Western countries for predicting the absolute risk of invasive breast cancer. Several other prospective studies looking at the accuracy of the GM were previously performed but these studies were done predominantly in Western populations: New York [16], Canada [17], Edinburgh [18], Malmo [19,20], Kopparberg and Ostergotland (Swedish Two-County) [21], Stockholm [22], Gothenburg [20,23], and Turkey [11]. Recruitment of Asian women into such trials conducted in Western populations has been rare and difficult. Relatively few publications have evaluated the GM in a population outside the US, and these were based primarily on case control data, which are more appropriately used for assessment of relative rather than absolute risk [9,10].

The SBCSP was a national prospective study of 28,235 predominantly Asian women. This is the only prospective trial conducted outside North America and Europe. Our results demonstrate, for the first time, an association between breast cancer incidence and the GM risk factors in an Asian population. Given the mature 10-year follow-up data of this study, the number of expected invasive breast cancer cases which was based on the GM was 1.85 times higher than the actual number observed in our local population. We conclude that the use of the GM considerably overestimates population risk for breast cancer incidence in Singaporean women who are not in a structured program of regular mammography screening. Given that our study was conducted in an Asian population, this result would be relevant to our Asian population and have a relevant impact on local chemoprevention studies and screening policies.

In our cohort, mammography was performed only once; thereafter, patients continued usual care. In Singapore, the incidence of breast cancer was 59.5 per 100,000 per year in the period from 2004 to 2008 [1]. From the International Agency for Research on Cancer's GLOBOCAN database, we obtained corresponding rates of 76 per 100,000 per year for the US [2]. Noting the differences in age-adjusted incidence rates between Singapore and the US, we considered the possibility that differences in distribution of the GM risk factors may account for this discrepancy if the GM included all relevant risk factors.

The use of screening would often result in a transient rise in numbers of pre-invasive and invasive lesions and a resultant fall in events in the years immediately following the period of screening because prevalent cases that were detected at initial screening were eliminated in subsequent years. This would result in fewer cases observed in the year immediately following the screening period and thus result in over-prediction. This was also observed in our cohort: the incidence of invasive breast cancer (per 10,000 woman-years) fell from an initial 37.5 to a mere 9.2 in the second year and 12.8 in the third year following screening. In the unscreened population, the invasive breast cancer incidence per 10,000 women remained relatively similar throughout all follow-up years after screening. However, the original model reported by Gail and colleagues [7] had computed the age-specific breast cancer incidence rates with inclusion of the first 3 years of the study. As such, we adhered to the same approach for model validation by including the first few years of follow-up in our data analysis.

Singapore is a small, dense, and highly electronically networked society. All Singaporeans have a unique identification number that enables accurate matching of electronic data with a central electronic cancer registry. Cancer notification is mandatory in Singapore and is managed through pathology departments. This enables near complete ascertainment of breast cancer incidence by linkage of SBCSP members with the cancer registry data.

There is minimal impact made on the results of our study despite the minor differences in coding of covariates in the SBCSP data when compared with the original GM. While the original GM includes the number of previous breast biopsies (0, 1, and 2 or more) and presence of atypical hyperplasia as risk factors, our analysis used an indicator of whether a woman had a previous breast biopsy (no, yes) in place of these factors for the SBCSP. These coding differences are unlikely to affect predictions meaningfully. In the NCI's SAS macro for estimating absolute breast cancer risk under GM, the relative risk of a women with one previous breast biopsy under GM (1.27 for those at least 50 years old) is very similar to that of a women with a previous breast biopsy (1.29 for those at least 50 years old). For unknown presence of atypical hyperplasia, the relative risk of all women with a previous breast biopsy done is multiplied by a factor of 1. In addition, the percentage of women with a previous breast biopsy in SBCSP is 5.2, which is lower than the 15% based on the BCDDP data used for the estimation of relative risk in the GM, further lowering the influence of this covariate on our study results.

The models overestimate risk in most of the categories. The discrepancy may arise from additional risk factors varying across populations not taken into account by the GM. In our study, the GM overestimates risk for women in whom age of menarche was at least 12 years, women who were more than 50 years old and who had no biopsy, or women without a first-degree relative affected and age at first live birth of 20 to 29 years, and these particular groups would benefit less from the use of mammography. It is of interest that the relative risks associated with family history are larger in our study as compared with those found in other studies performed in Western populations. This is especially so for women with more than one affected first-degree relative with breast cancer, in whom the larger relative risks could be due to the small number of such women in our study. Matsuno and colleagues [15] also reported breast cancer risks in Asian-American patients. In that study, the relative risks associated with family history were not larger in an Asian-American population when compared with the original GM. However, the study by Matsuno and colleagues is much smaller than ours, and the subjects are derived from an immigrant Asian population in the West. Hence, our study observation is likely to be representative and meaningful.

The good performance of the IT-GM and the IT1-GM using independent data of Florence-EPIC (Florence-European Prospective Investigation into Cancer and Nutrition) indicates that the data from the Italian Multicentre Case Control Study of Diet and Breast Cancer might be useful for revising the GM to include additional risk factors, particularly modifiable risk factors, such as dietary consumption patterns. In principle, the case control data used in this study, together with cancer registry data, can be used to construct such models of absolute risk, and the current findings encourage us to do so.

The extent of over-prediction of number of invasive breast cancers by GM within 10 years from screening (E/O ratio = 1.85) was smaller than that for the 5-year period (E/O ratio = 2.51). There are a few possible reasons for this decrease in E/O ratio. One possible explanation is the increase in breast cancer incidence with age. This results in larger numbers of breast cancer diagnosed in an older population. A larger incidence of breast cancer would better approximate the risks of breast cancer incidence calculated by the GM. In addition, it is possible that, over time, an increased proportion of women adopted regular mammographic screening, thereby resulting in an increase of breast cancer detection and consequent narrowing of our E/O ratio as calculated by using the GM. There was a significant lack of fit between the observed and expected breast cancer numbers based on GM for the SBCSP study cohort. By age cohort, females who were 50 to 59 years old appeared to derive a better benefit from a one-time screening mammography in comparison with females who were 60 to 64 years old.

The Matsuno models gave a breast cancer number that was closer to the observed count as compared with the Caucasian-American GM model, but overestimation remained at 5-year follow-up (Tables 3, 4, 5 and 6). Based on the Matsuno models, the 5-year E/O ratio was 1.43. This was much lower than the corresponding ratio of 2.51 based on GM. For the 10-year prediction period, the E/O ratio was 0.99 based on the Matsuno models and 1.85 based on GM. There was no significant difference between the expected count and observed count of breast cancers by presence of previous biopsy, 5-year age at screening age group, and predicted risk quintile groups when the predictions are based on the Matsuno models. Possible reasons to explain the presence of an over-prediction at 5 years and its subsequent disappearance at 10 years are multifactorial.

### Calibration of the Gail model

Decarli and colleagues [24] pointed out, in a recent article establishing the validity of the GM in a Southern European population, that the GM may be well calibrated for Western European populations in which mammographic screening is common but that its applicability remains uncertain in populations in which screening is less frequent. Although the good calibration of the GM in the Florence-EPIC cohort suggests that it may be a useful model for predicting risk in other Western European populations in which mammographic screening is common, our results suggest that, at the minimum, the GM may be less useful in our prospective study population in which screening is less frequent.

Based on rapid fertility and lifestyle changes in the Singapore population (particularly in the 1970s), an age-cohort model is thought to provide possibly the best prediction for breast cancer trends seen [25]. In our study population, the GM provided the best predictions for individuals from the 50- to 54-year age group (E/O ratio = 1.52, 95% CI 1.29 to 1.80). This may be compared with the setting of older women who were 60 to 64 years old (E/O ratio = 2.54, 95% CI 2.10 to 3.07), in whom the model significantly over-predicts the risk by up to three times. There has been a steady increase in breast cancer incidence in Singapore to match that of Western countries [25]. The reason for this trend is multifactorial and includes increased adoption of Western lifestyle and changes in fertility.

### Risk of over-diagnosis

Over-diagnosis is used to describe a condition that is diagnosed but that otherwise would not cause symptoms or go on to cause death. This occurs when the cancer either does not progress or grows at such a slow rate that the individual succumbs to other comorbidities. Given the increasing prevalence of cancer screening together with the improvements in screening technologies and interventions, the issue of over-diagnosis is now more evident. The 15-year follow-up results of the Malmo mammographic screening trial suggest that the risk of over-diagnosis of a mammographically detected cancer is 24% [26]. While early detection of invasive cancers aids in the treatment and improved survival for breast cancer, the role of early treatment of DCIS has not been well studied [27]. Four of the five large trials that found breast cancer mortality reductions, however, found no overall mortality reductions [28]. In addition, DCIS has been reported to have excellent survival [17], and it has been suggested that one in three 'invasive' cancers detected by mammography is non-threatening [29].

For patients, early detection adds to the increased risks of procedures such as breast biopsies as well as the risks of unnecessary treatment and medical expenses. Over-diagnosis also adds to patient anxiety and has social implications. A thorough discussion between clinician and patient should be carried out to ensure that the patient understands the implications and limitations of screening prior to proceeding with it. Strategies to reduce the risk of over-diagnosis would include a review of the current risk factors in our local population and a refinement of our risk prediction models to allow us to better advise our patients with regard to the need for screening.

Much of our data are based on information obtained from our national cancer and diseases registry, which has excellent coverage. However, it is true that certain individuals who had migrated from the country and changed citizenship would subsequently be lost to follow-up on such a national database. However, the numbers of migrants remain small and should not affect our results significantly. It should be noted that our study represents the only systematic one-time mammography screening study performed to date and differs from other studies that were conducted on the basis of annual/biennial mammograms. With increased ascertainment of incidental tumors, the incidence of breast cancer would be higher with annual screening as compared with a single screening mammogram.

In summary, we report long-term results from a large national prospective breast cancer mammographic screening study from Singapore, the only one conducted outside North America and Europe. Our study validates the GM risk factors individually but demonstrates that the GM overestimates individual risk for breast cancer in the setting of a developed country in Asia. With industrialization and its accompanying lifestyle shifts, Asia has witnessed a dramatic rise of breast cancer incidence, representing a huge global burden. Consequently, it has become imperative to evaluate local breast cancer epidemiology in order to validate existing models of breast cancer risk. With our results, future work should focus on the development of appropriately calibrated models for better prediction of risk, which would benefit individual counseling and cancer prevention research.

## Abbreviations

AABCS: Asian American Breast Cancer Study; BCDDP: Breast Cancer Detection Demonstration Project; CI: confidence interval; DCIS: ductal carcinoma *in situ*; E: expected number of breast cancer cases; E/O: ratio of expected to observed number of breast cancer cases; EPIC: European Prospective Investigation into Cancer and Nutrition; GM: Gail model; NCI: National Cancer Institute; O: observed number of breast cancer cases; SBCSP: Singapore Breast Cancer Screening Project; SEER: Surveillance: Epidemiology: and End Results.

## Competing interests

The authors declare that they have no competing interests.

## Authors' contributions

MHT participated in the design and coordination of the study and helped to perform the statistical analysis and write the manuscript. PA participated in the design and coordination of the study. WYC, WSO and NQJL helped to perform the statistical analysis and write the manuscript. GHH, PHT, CSW, KSC, and KYC reviewed the manuscript and provided advice. All authors read and approved the final manuscript.
